# Supplementary material for: Tead1a Initiates Transcriptional Priming Through the TEAD1a/YAP‐Notch1‐Spi1/Cebpα Axis to Promote Neutrophil Fate
Source: Adv Sci (Weinh). 2025 Aug 28;12(41):e05441. doi: 10.1002/advs.202505441 (PMC12591127; doi:10.1002/advs.202505441)
Supplement: Supplementary file 4 — Supplementary Dataset [file ADVS-12-e05441-s004.zip › 3-5.zip!/3-5/02-5.cluster/cluster13/Enrich/ego_all.pdf]

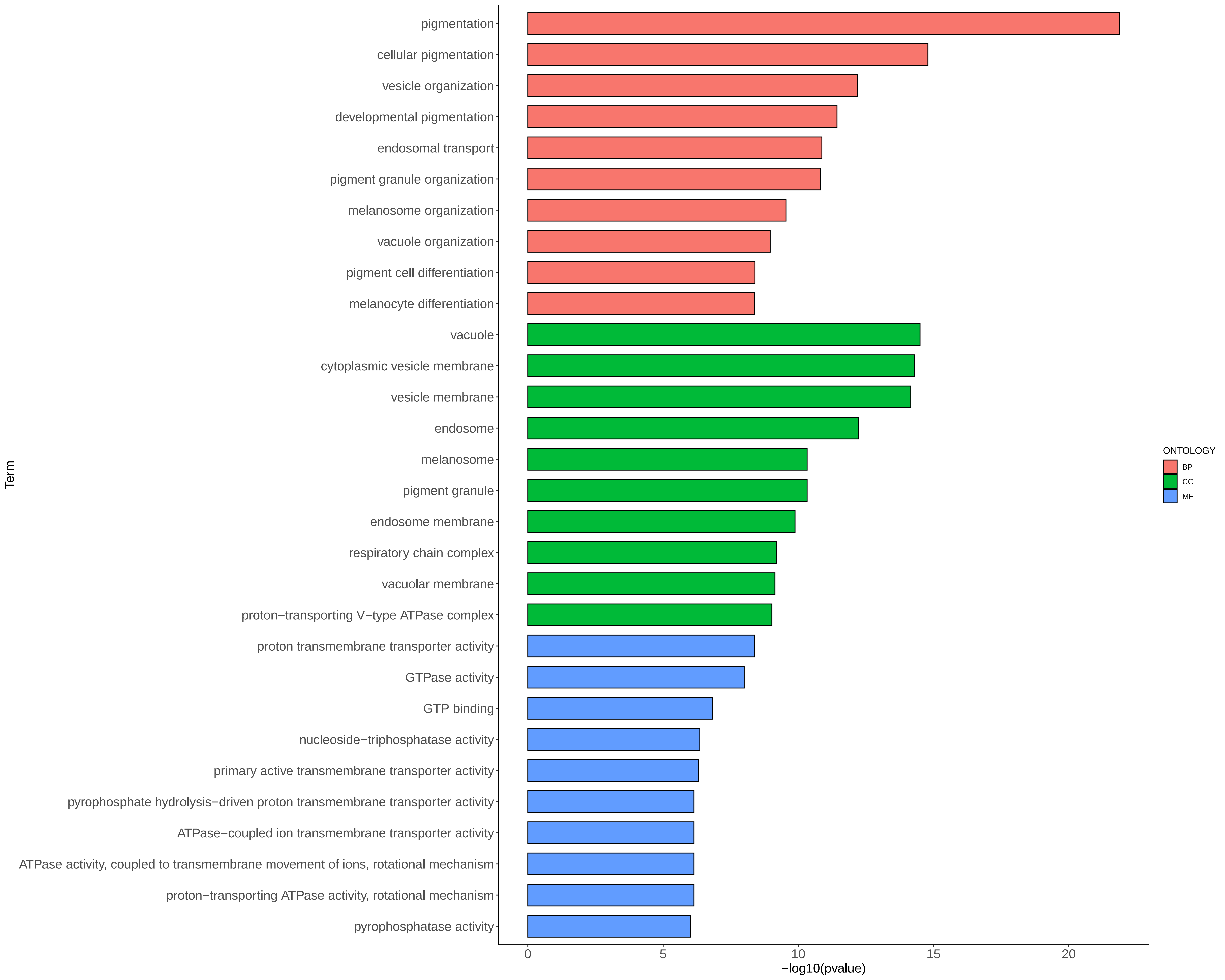
pigmentation
cellular pigmentation
vesicle organization
developmental pigmentation
endosomal transport
pigment granule organization
melanosome organization
vacuole organization
pigment cell differentiation
melanocyte differentiation
vacuole
cytoplasmic vesicle membrane
vesicle membrane
endosome
melanosome
pigment granule
endosome membrane
respiratory chain complex
vacuolar membrane
proton−transporting V−type ATPase complex
proton transmembrane transporter activity
GTPase activity
GTP binding
nucleoside−triphosphatase activity
primary active transmembrane transporter activity
pyrophosphate hydrolysis−driven proton transmembrane transporter activity
ATPase−coupled ion transmembrane transporter activity
ATPase activity, coupled to transmembrane movement of ions, rotational mechanism
proton−transporting ATPase activity, rotational mechanism
pyrophosphatase activity
Term
0
5
10
15
20
−log10(pvalue)
ONTOLOGY
BP
CC
MF
